# Dietary PUFA Preferably Modify Ethanolamine-Containing Glycerophospholipids of the Human Plasma Lipidome

**DOI:** 10.3390/nu14153055

**Published:** 2022-07-26

**Authors:** Christine Dawczynski, Johannes Plagge, Gerhard Jahreis, Gerhard Liebisch, Marcus Höring, Claudine Seeliger, Josef Ecker

**Affiliations:** 1 Institute of Nutritional Sciences, Friedrich Schiller University Jena, 07743 Jena, Germany; christine.dawczynski@uni-jena.de (C.D.); gerhard.jahreis@uni-jena.de (G.J.); 2 Research Group Lipid Metabolism, ZIEL Institute for Food & Health, Technical University of Munich, Gregor-Mendel-Str. 2, 85354 Freising, Germany; johannes.plagge@tum.de (J.P.); claudine.seeliger@tum.de (C.S.); 3 Institute of Clinical Chemistry and Laboratory Medicine, University Hospital Regensburg, 93053 Regensburg, Germany; gerhard.liebisch@ukr.de (G.L.); marcus.hoering@ukr.de (M.H.)

**Keywords:** lipidomics, phosphatidylethanolamine, plasma, plasmalogen, PUFA, vegetable oil

## Abstract

The content of polyunsaturated fatty acids (PUFA) in complex lipids essentially influences their physicochemical properties and has been linked to health and disease. To investigate the incorporation of dietary PUFA in the human plasma lipidome, we quantified glycerophospholipids (GPL), sphingolipids, and sterols using electrospray ionization coupled to tandem mass spectrometry of plasma samples obtained from a dietary intervention study. Healthy individuals received foods supplemented with different vegetable oils rich in PUFA. These included sunflower, linseed, echium, and microalgae oil as sources of linoleic acid (LA; FA 18:2 *n*-6), alpha-linolenic acid (ALA; FA 18:3 *n*-3), stearidonic acid (SDA; FA 18:4 *n*-3), and docosahexaenoic acid (DHA; FA 22:6 *n*-3). While LA and ALA did not influence the species profiles of GPL, sphingolipid, and cholesteryl ester drastically, SDA and DHA were integrated primarily in ethanolamine-containing GPL. This significantly altered phosphatidylethanolamine and plasmalogen species composition, especially those with 38–40 carbons and 6 double bonds. We speculate that diets enriched with highly unsaturated FA more efficiently alter plasma GPL acyl chain composition than those containing primarily di- and tri-unsaturated FA, most likely because of their more pronounced deviation of FA composition from typical western diets.

## 1. Introduction

Nature features a huge diversity of lipid molecules, including glycerophospholipid(s) (GPL), sphingolipid(s) (SL), and sterols, which are major cell membrane components [1,2]. The fatty acyl composition of GPL critically influences the physicochemical properties of lipid bilayers. The proportions of unsaturated fatty acyls determine membrane viscosity because the molecular shapes of acyl moieties affect lipid packing [3]. In contrast to saturated acyl chains packed with higher densities, forming non-fluid gel phases, monounsaturated fatty acyls with kinked shapes reduce packing density and increase fluidity. Polyunsaturated fatty acyls have extraordinary flexibility since they can switch easily between different conformations, allowing adaption to different membrane shapes. GPL containing docosahexaenoic acid (DHA; FA 22:6 *n*-3) facilitate membrane shaping and fission of dynamin and endophilin involved in endocytic vesicle formation [4]. Polyunsaturated fatty acid(s) (PUFA) are found at very high concentrations in the GPL of synaptic vesicles [5]. Neuron axon tips are enriched with DHA-phosphatidylcholine (PC) [6] and the brain with PUFA-phosphatidylethanolamine (PE) and -phosphatidylserine [7,8]. Interestingly, *n*-3 unsaturated GPL provide more flexibility to membranes than *n*-6, which might explain why the relative amounts of *n*-3 and *n*-6 are so important in the membranes of brain cells [9]. The *n*-3/*n*-6 ratio, in addition to its relevance for membrane biophysical properties, is also key for the generation of inflammatory eicosanoids and anti-inflammatory specialized pro-resolving mediators critical for numerous cellular processes, including cell death/survival and inflammatory disease [10,11]. Higher intake of *n*-3 PUFA correlates with a lower incidence of chronic inflammatory diseases, including cardiovascular disease (CVD) [12]. Intervention trials using eicosapentaenoic acid (EPA, FA 20:5 *n*-3) and DHA indicate lower mortality of CVD patients and a significant inverse linear dose–response relationship with pathological outcomes.

The impact of dietary PUFA on the human lipidome is complex, particularly because lipid species composition (within organs and cell types) is conserved, as it is custom-fitted to its biological function [13,14,15,16,17]. Disturbances are equalized since the mammalian lipidome is extremely dynamic to ensure cell functionality [3,18]. To determine if and how dietary PUFA are integrated into endogenous complex lipids, we here analyzed GPL, sphingolipid (SL), and sterol composition of plasma samples obtained from a randomized, double-blind crossover dietary intervention study, where healthy individuals received foods supplemented with different vegetable oils rich in PUFA [19].

## 2. Materials and Methods

### 2.1. Study Design and Participants

**Figure 1:**
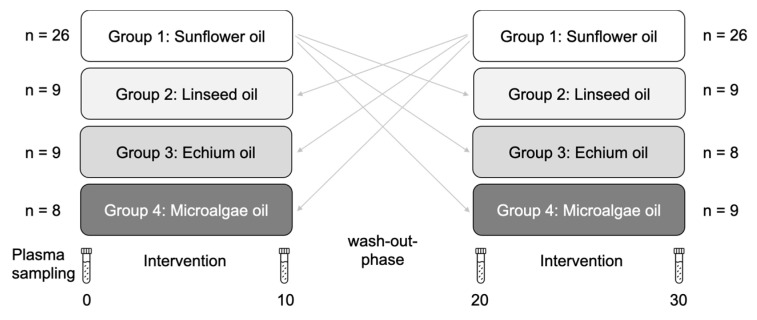
Study design and blood sampling.

A total of 59 volunteers (39 f; 20 m) between 25 and 75 years old recruited around Jena (Germany) were enrolled in the randomized, placebo-controlled, double-blind, cross-over study (Figure 1) [19]. The 10-week (wk) intervention periods were separated by a 10-wk washout phase. Participants were in close contact with the study team to ensure compliance. The intake of lipid-lowering medications and glucocorticoids were exclusion criteria as well as taking dietary supplements (e.g., fish oil capsules, vitamin E), gastrointestinal or metabolic diseases (e.g., diabetes mellitus, hyperthyroidism or hypothyroidism, hypercholesteremic patients with familial previous impacts), daily alcohol abuse, and known allergies or foodstuff indigestibility. Written informed consent was obtained from all subjects involved in the study (ClinicalTrials.gov. Identifier: NCT01437930). The study foods (sausage, 60 g/d; bread rolls, 100 g/d; milk powder, 20 g/d; crispy wafer with chocolate spread, 35 g/d) were fortified with (A) sunflower oil (62% linoleic acid, LA; FA 18:2 *n*-6) or (B) linseed oil (53% alpha-linolenic acid, ALA; FA 18:3 *n*-3) or (C) oil from Echium plantagineum (31% ALA and 11% stearidonic acid, SDA; FA 18:4 *n*-3) or (D) microalgae oil from Schizochytrium sp. used as a powder (17% docosahexaenoic acid, DHA; FA 22:6 *n*-3). Linseed oil and sunflower oil were provided by PPM e.V. Germany, Magdeburg, and echium oil and microalgae powder were obtained from HARKE Nutrition, Mühlheim an der Ruhr, Germany.

Participants consumed (per day) approximately (A) 20.0 g sunflower oil with a mean content of 10.0 g LA or (B) 20 g linseed oil containing 7.4 g ALA or (C) 20.0 g echium oil with 4.8 g ALA and 1.6 g SDA or (D) 12.0 g microalgae oil powder with 1.6 g DHA. The FA composition of the different vegetable oils was described previously [19]. Comparable dosages of the study oils/powder resulting in different intakes of ALA, SDA, LA, and DHA were used to ensure isocaloric interventions. At the beginning and end of each intervention period, blood samples were drawn by venipuncture after a 12 h overnight fast, and body weight and blood pressure were measured. Baseline characteristics of study participants are shown in Table 1.

The Ethics Committee of the Friedrich Schiller University of Jena approved the study (2610-07/09) and it was registered on clinicaltrials.gov. (NCT01437930).

### 2.2. Lipidomics

Lipids were quantified by direct flow injection electrospray ionization tandem mass spectrometry (ESI-MS/MS) in positive ion mode using the analytical setup and strategy described previously [20,21]. Lipid extraction was performed according to the method of Bligh and Dyer [22] in the presence of non-naturally occurring lipid species as internal standards. The following lipid species were added as internal standards: PC 14:0/14:0, PC 22:0/22:0, PE 14:0/14:0, PE 20:0/20:0 (di-phytanoyl), PI 17:0/17:0, LPC 13:0, LPC 19:0, Cer 18:1;O2/14:0, Cer 18:1;O2/17:0, D7-FC, CE 17:0, and CE 22:0.

A fragment ion of *m/z* 184 was used for phosphatidylcholine (PC), sphingomyelin (SM) [20], and lysophosphatidylcholine (LPC) [23]. Neutral loss fragments were used for the following lipid classes: Phosphatidylethanolamine (PE) and phosphatidylinositol (PI) with a loss of 141 and 277, respectively [24]. PE-based plasmalogens (PE P) were analyzed according to the principles described by Zemski-Berry [25]. Sphingosine-based ceramides (Cer) were analyzed using a fragment ion of *m/z* 264 [26]. Free cholesterol (FC) and cholesteryl ester (CE) were quantified using a fragment ion of *m/z* 369 after selective derivatization of FC [21]. Quantification was achieved using two non-naturally occurring internal standards (IS) for each lipid class (except for PI, SM was calculated using PC IS, and PE-based plasmalogens were calculated using PE IS) and calibration lines generated by the standard addition of a number of naturally occurring species to the plasma. Calibration lines were generated for the following naturally occurring species: PC 34:1, 36:2, 38:4, 40:0 and PC O-16:0/20:4; SM 18:1;O2/16:0, 18:1, 18:0; LPC 16:0, 18:1, 18:0; PE 34:1, 36:2, 38:4, 40:6 and PE P-16:0/20:4; Cer 18:1;O2/16:0, 18:0, 20:0, 24:1, 24:0; FC, CE 16:0, 18:2, 18:1, 18:0. Deisotoping and data analyses for all lipid classes were performed by self-programmed Excel macros as previously described [20]. Lipid species were annotated according to the latest proposal for shorthand notation of lipid structures that are derived from mass spectrometry [27]. GPL species annotation was based on the assumption of even-numbered carbon chains only. The annotation of the SM species is based on the assumption that a sphingoid base with two hydroxyl groups is present.

Sample material was not available/suitable for the lipidomic analysis from all study participants. Thus, the *n*-values in the analyses figures may not match the number of study participants shown in Figure 1.

### 2.3. Lipidomic Data Processing and Statistical Analyses

The lipid species profiles of lipid classes shown as “% of total” in the bar plots were calculated from molar concentrations. For volcano plot analyses, only the lipid species with an average contribution > 1% per lipid class were included. Missing or zero values were replaced by 1/5 of the smallest non-zero value of each lipid species and data were log2 transformed. A paired t-test was used to test for significant differences between “before” and “at the end of the 10 wk intervention period”. To correct for multiple testing, the FDR was controlled at 0.01 using the Benjamini–Hochberg method. Statistical analyses were performed with Microsoft Excel 2016.

## 3. Results and Discussion

### 3.1. Lipidomics and Data Analysis

A quantitative lipidomic analysis of plasma samples was performed using direct infusion electrospray ionization coupled to tandem mass spectrometry (ESI-MS/MS) comprising: (i) Glycerophospholipids (GPL: phosphatidylcholine, PC; lyso-PC, LPC; phosphatidylethanolamine, PE; PE-based plasmalogens, PE P; phosphatidylinositol, PI); (ii) Sphingolipids (SL: ceramide, Cer; sphingomyelin, SM) and (iii) sterols (free cholesterol, FC; cholesteryl ester; CE). In total, 298 species were quantified in plasma samples. CE was the dominating lipid class, followed by PC and FC (Figure S1). To test whether vegetable oils affect lipidomic profiles, plasma samples at baseline were compared with those obtained after 10 weeks (wks) of dietary intervention. Lipid species were considered significantly different between baseline and 10 wks if their p-value passed the multiple-testing correction threshold (FDR < 0.01). The data analysis was applied to species profiles of lipid classes that were calculated from molar concentrations since the calculation of lipid profiles normalizes the data and minimizes inter-individual differences and variations. Similarly, this data analysis strategy was recently successfully applied to investigate the lipidomes of tumor samples from colorectal cancer patients [17,28].

### 3.2. Linoleic Acid from Sunflower Oil Associates with Saturated Fatty Acids in Cholesteryl Ester and Glycerophospholipids

**Figure 2:**
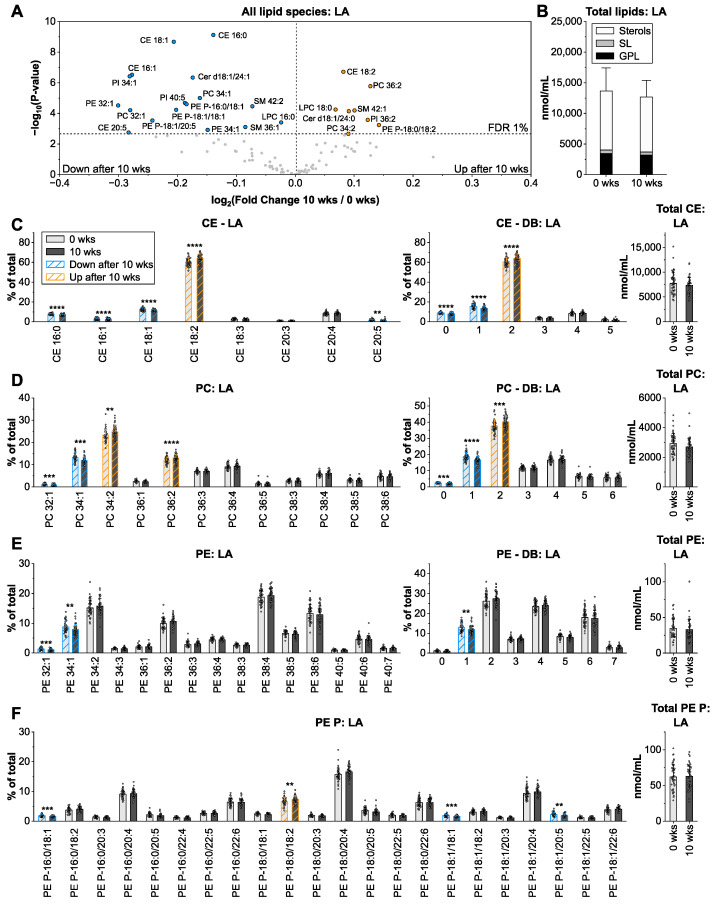
Impact of sunflower oil intervention on the human plasma lipidome. (**A**) Volcano plots showing a significantly different lipid species after 10 weeks (wks) of intervention. (**B**) Total concentration and distribution of all analyzed lipids. (**C**–**F**) Lipid species profiles, double bond (DB) composition in FA moieties, and total lipid levels for cholesteryl ester (CE), phosphatidylcholine (PC), phosphatidylethanolamine (PE), and PE-based plasmalogens (PE P). Shown are means ± SD of the lipid species with an average contribution > 1% from *n* = 45. ** *p* < 0.01, *** *p* < 0.001, **** *p* < 0.0001.

Dietary intervention with food enriched in sunflower oil (62% linoleic acid, LA; FA 18:2 *n*-6) did not alter total lipid amounts (Figure 2B). An analysis of the lipid species pattern revealed marginal (fold change: −1.2 to +1.1), but highly significant alterations after 10 wks, primarily in CE and GPL (Figure 2A). Proportions of di-unsaturated lipid species, including CE 18:2, PC 34:2, PC 36:2, PE P-18:0/18:2, and PI 36:2 were higher after dietary intervention, while saturated and monounsaturated lipid species were lowered (Figure 2D–F and Figure S2). Interestingly, higher unsaturated GPL, such as PC 38:6 and PE 38:6, were unchanged, suggesting that FA 18:2 *n*-6 rather associates with FA 16:0 and FA 18:0 than with 20:4. Together, we conclude that LA primarily enriches in plasma CE and GPL. We have previously shown that linoleic acid is preferentially incorporated into neutral lipids including CE in human macrophages [29]. Tracing the fate of [U13C]-labeled FA 18:2 *n*-6 over 24 h in healthy subjects, Hodson and colleagues found elevated levels of LA in plasma CE and GPL [30].

### 3.3. Alpha-Linolenic Acid from Linseed Oil Lowers Total Lipid Levels

**Figure 3:**
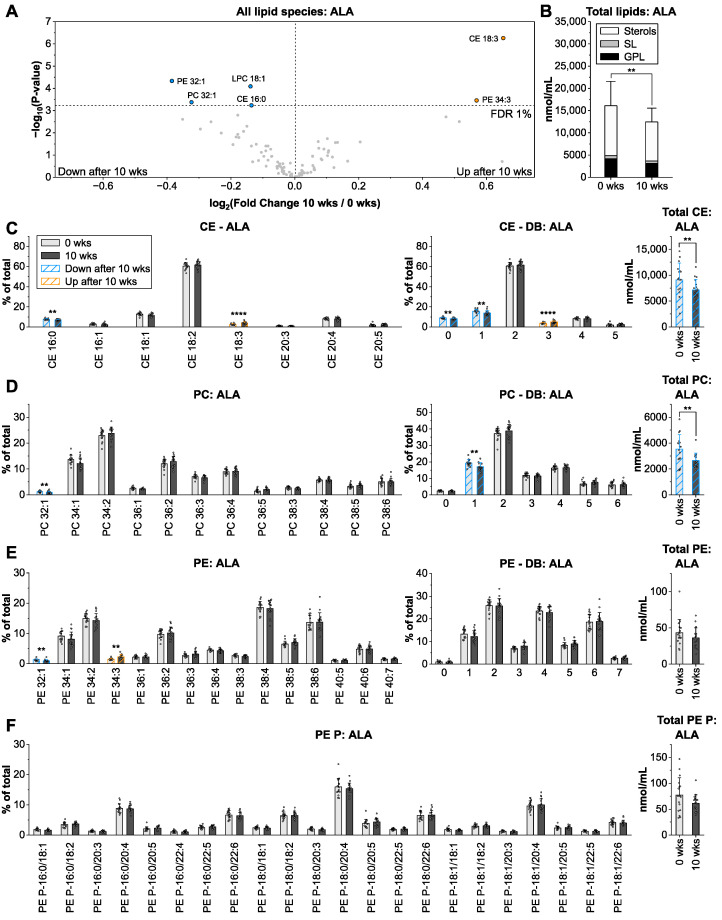
Impact of linseed oil intervention on the human plasma lipidome. The arrangement of panels (**A**–**F**) is identical to Figure 2. Shown are means ± SD of lipid species with an average contribution > 1% from *n* = 17. ** *p* < 0.01, **** *p* < 0.0001.

Supplementation with linseed oil (53% alpha-linolenic acid, ALA; FA 18:3 *n*-3) lowered total lipid concentrations by 23%, including GPL, SL, and sterols (Figure 3B). The fraction of CE 18:3 was 1.6-fold higher at 10 wks compared to baseline, while that of CE 16:0 was reduced (Figure 3A,C). In the group of GPL, solely PE 34:3 proportions were elevated after 10 wks. Profiles of Cer and SM were unaltered (Figure 3D–F and Figure S3). Compared to the sunflower oil group, fewer lipid species were altered (6 vs. 25), but the magnitudes of change were higher (Fold change: −1.3 to +1.6) (Figure 3A). Moreover, linseed oil supplementation did not alter the contents of highly unsaturated GPL with DB > 5, suggesting that FA 18:3 *n*-3 is preferably inserted in GPL acyl combinations with saturated FA, as observed for LA, and that it is not significantly metabolized to FA 22:6 *n*-3. It was previously reported that the formation of DHA from ALA is negligible (<1%) in humans as well as independent of the dietary PUFA content [31,32].

### 3.4. Stearidonic Acid from Echium Oil Enriches in Glycerophospholipids with 4–5 Double Bonds

**Figure 4:**
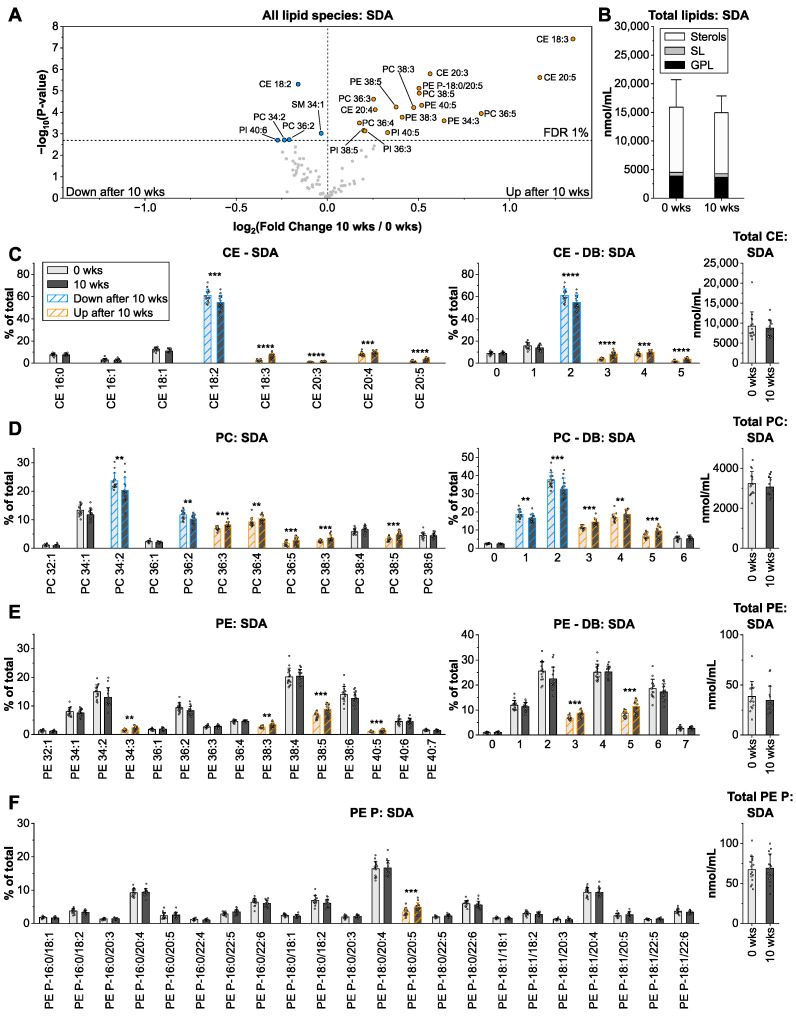
Impact of echium oil intervention on the human plasma lipidome. The arrangement of panels (**A**–**F**) is identical to Figure 2. Shown are means ± SD of lipid species with an average contribution > 1% from *n* = 15. ** *p* < 0.01, *** *p* < 0.001, **** *p* < 0.0001.

Although the intervention with echium oil (31% ALA and 11% stearidonic acid, SDA; FA 18:4 *n*-3) did not alter total lipid concentrations, lipid species composition was more drastically affected than observed for sunflower and linseed oils (Figure 4A,B). The proportions of 22 lipid species were significantly altered with fold changes between −1.2 and +2.5-fold. After 10 wks of intervention, the GPL pattern shifted towards species with 4–5 double bonds (Figure 4C–F and Figure S4). PC 36:4, PC 36:5, PC 38:5, PE 38:5, PE 40:5, PI 38:5, and PI 40:5 increased up to 1.8-fold, suggesting that SDA associates with saturated and monounsaturated FA, such as FA 18:0, FA 18:1, FA 20:1, FA 22:1.

In contrast to ALA, SDA is more efficiently converted to EPA [32], explaining the 1.4-fold elevated PE P-18:0/20:5 levels. However, its conversion to DHA is limited [32] confirmed by the unchanged fraction of GPL with DB > 5. ALA, which is also a major component of echium oil with 31%, is incorporated in triunsaturated CE and GPL species (CE 18:3, PC 36:3, PC 38:3; PE 34:3, PE 38:3, and PI 36:3) as observed in the linseed oil group.

### 3.5. Docosahexaenoic Acid from Microalgae Oil Changes the Species Profiles of Ethanolamine-Containing Glycerophospholipids

**Figure 5:**
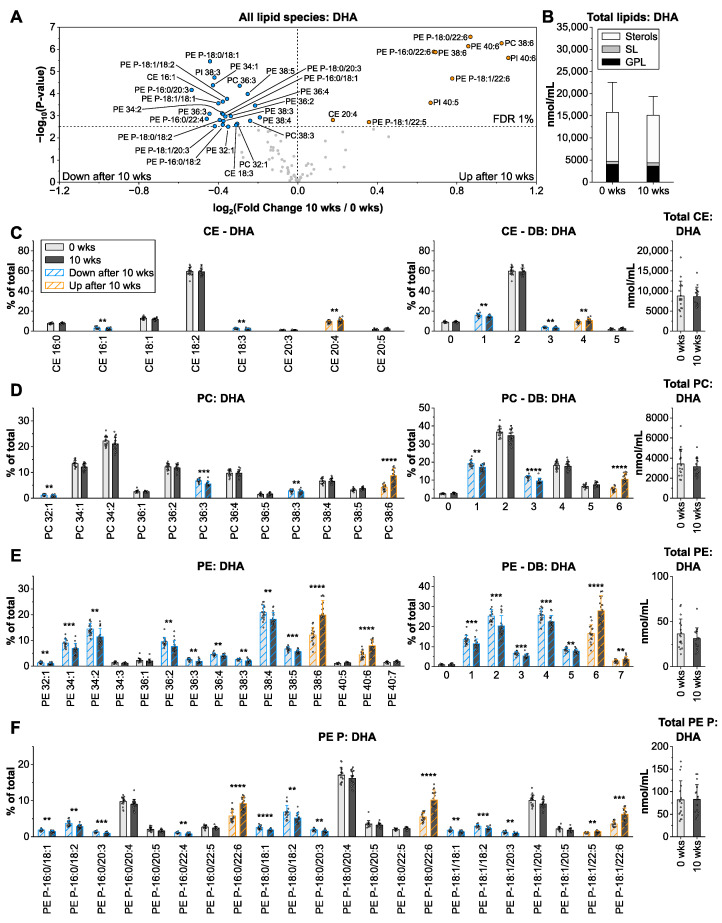
Impact of microalgae oil intervention on the human plasma lipidome. The arrangement of panels (**A**–**F**) is identical to Figure 2. Shown are means ± SD of lipid species with an average contribution > 1% from *n* = 17. ** *p* < 0.01, *** *p* < 0.001, **** *p* < 0.0001.

Microalgae oil, as a plant-based source of highly unsaturated *n*-3 PUFA, was chosen, as the intake of sea fish is restricted due to limited resources, overfishing, and sensory aspects. In general, the establishment of alternative plant-derived dietary *n*-3 PUFA sources is of urgent need. Amongst all groups, microalgae oil (17% docosahexaenoic acid, DHA; FA 22:6 *n*-3) most significantly affected the plasma lipidome. 36 species, including 26 ethanolamine-containing GPL, were altered with fold changes from −1.5 to +2.1 (Figure 5A).

DHA was incorporated into FA 16:0, FA 18:0, and FA 18:1 containing PE P, as well as in PC, PE, and PI species with 38–40 carbons and 6 double bonds (Figure 5D–F and Figure S5). Shorter and less unsaturated GPL with 1–3 double bonds, including PC/PE 32:1, PC 34:2, PC/PE 36:3, and PC/PE 38:3 were reduced after 10 wks of microalgae oil intervention. Saturated GPL and SL as well as total lipid levels (Figure 5B and Figure S5) remained unchanged. DHA from fish oil was previously shown to elevate plasma PUFA-PE concentrations [33,34] and to incorporate in plasma PC already after 1–2 h after intake in healthy humans [35]. In agreement with our results, applying [13C]-labeled DHA in young adults demonstrated that it accumulates more efficiently in plasma PE than PC [36]. This is most likely due to the pronounced specificity of liver ethanolamine phosphotransferase for 1-saturated, 2-docosahexaenoyl-glycerol, in contrast to choline phosphotransferase [37]. We also hypothesize that PE and PE P simply provide a higher capacity for the enrichment of highly unsaturated FA since their acyl chains are generally longer and more unsaturated than those of PC.

## 4. Conclusions

Together, we show that PUFA from vegetable oils incorporate into plasma CE and GPL. We hypothesize that they are evenly distributed into endogenous lipids to minimize alterations and sustain the existing and optimized lipid composition. Because the fraction of higher unsaturated PUFA in typical western diets is low, SDA and DHA induce more pronounced alterations of acyl chain composition of PE, PE P, and PC by shifting it towards long and highly unsaturated species. Plasma PUFA are transported to peripheral tissues and become part of cell membranes, which influence membrane biophysics, such as GPL acyl chain flexibility as well as PUFA-derived inflammation-related lipid messenger profiles. We previously showed that regular consumption of foods enriched with echium oil and microalgae oil leads to health-promoting anti-inflammatory effects [38,39].

## Figures and Tables

**Table 1:** Baseline characteristics of the study population.

*n* = 59 (39 f, 20 m)		Mean ± SD	Min	Max
Age	years	54 ± 12	27	74
Body mass index	kg/m^2^	28.6 ± 5.3	20.0	43.8
Systolic blood pressure	mmHg	146.3 ± 20.0	105.0	192.0
Diastolic blood pressure	mmHg	89.7 ± 10.9	60.0	117.0
Pulse	beats per minute	71.4 ± 13.0	53.0	120.0
Triacylglycerol	mmol/L	2.18 ± 1.40	0.70	7.36
Total cholesterol	mmol/L	5.74 ± 1.06	3.13	8.29
LDL cholesterol	mmol/L	3.61 ± 0.96	1.64	6.00
HDL cholesterol	mmol/L	1.09 ± 0.37	0.54	2.32
LDL/HDL		3.60 ± 1.18	0.70	7.71
hs-CRP	mg/L	3.64 ± 3.74	0.50	14.70

## Data Availability

Lipidomic data analyzed in this study are included in this article as figures.

## Supplementary Materials

The following supporting information can be downloaded at: https://www.mdpi.com/article/10.3390/nu14153055/s1, Figure S1: Lipid class composition of plasma samples; Figure S2: Impact of sunflower oil intervention on plasma PI, LPC, SM and Cer profiles; Figure S3: Impact of linseed oil intervention on plasma PI, LPC, SM and Cer profiles; Figure S4: Impact of echium oil intervention on plasma PI, LPC, SM and Cer profiles; Figure S5: Impact of microalgae oil intervention on plasma PI, LPC, SM and Cer profiles.

## Abbreviations

ALA, alpha-linolenic acid; CE, cholesteryl ester; Cer, ceramide; CVD, cardiovascular disease; DB, double bond; DHA, docosahexaenoic acid; EPA, eicosapentaenoic acid; ESI-MS/MS, electrospray ionization tandem mass spectrometry; FA, fatty acid; FC; free cholesterol; FDR, false discovery rate; GPL, glycerophospholipid; HDL, high-density lipoprotein; hs-CRP, high-sensitivity C-reactive protein; IS, internal standard; LA, linoleic acid; LDL, low-density lipoprotein; LPC, lysophosphatidylcholine; *m*/*z*, mass-to-charge ratio; PC, phosphatidylcholine; PE, phosphatidylethanolamine; PE P, PE-based plasmalogen; PI, phosphatidylinositol; PUFA, polyunsaturated fatty acid; SDA, stearidonic acid; SL, sphingolipid; SM, sphingomyelin.
