# Supplementary material for: Dietary PUFA Preferably Modify Ethanolamine-Containing Glycerophospholipids of the Human Plasma Lipidome
Source: Nutrients. 2022 Jul 26;14(15):3055. doi: 10.3390/nu14153055 (PMC9332067; doi:10.3390/nu14153055)
Supplement: Supplementary file 1 [file nutrients-14-03055-s001.zip › nutrients-1794186-supplementary.pdf]

Supplementary Materials

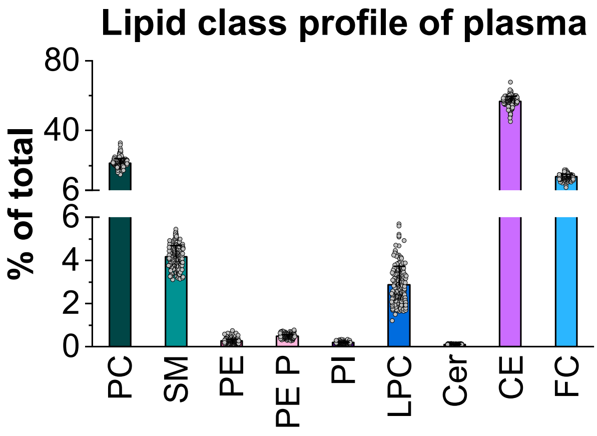

Figure S1. Lipid class profile in plasma. Shown are means ± SD from n = 188. PC: Phosphatidylcholine, SM: Sphingomyelin, PE: Phosphatidylethanolamine, PE P: PE-based plasmalogens, PI: Phosphatidylinositol, LPC: Lysophosphatidylcholine, Cer: Ceramides, CE: Cholesteryl ester, FC: Free cholesterol.

Figure S2

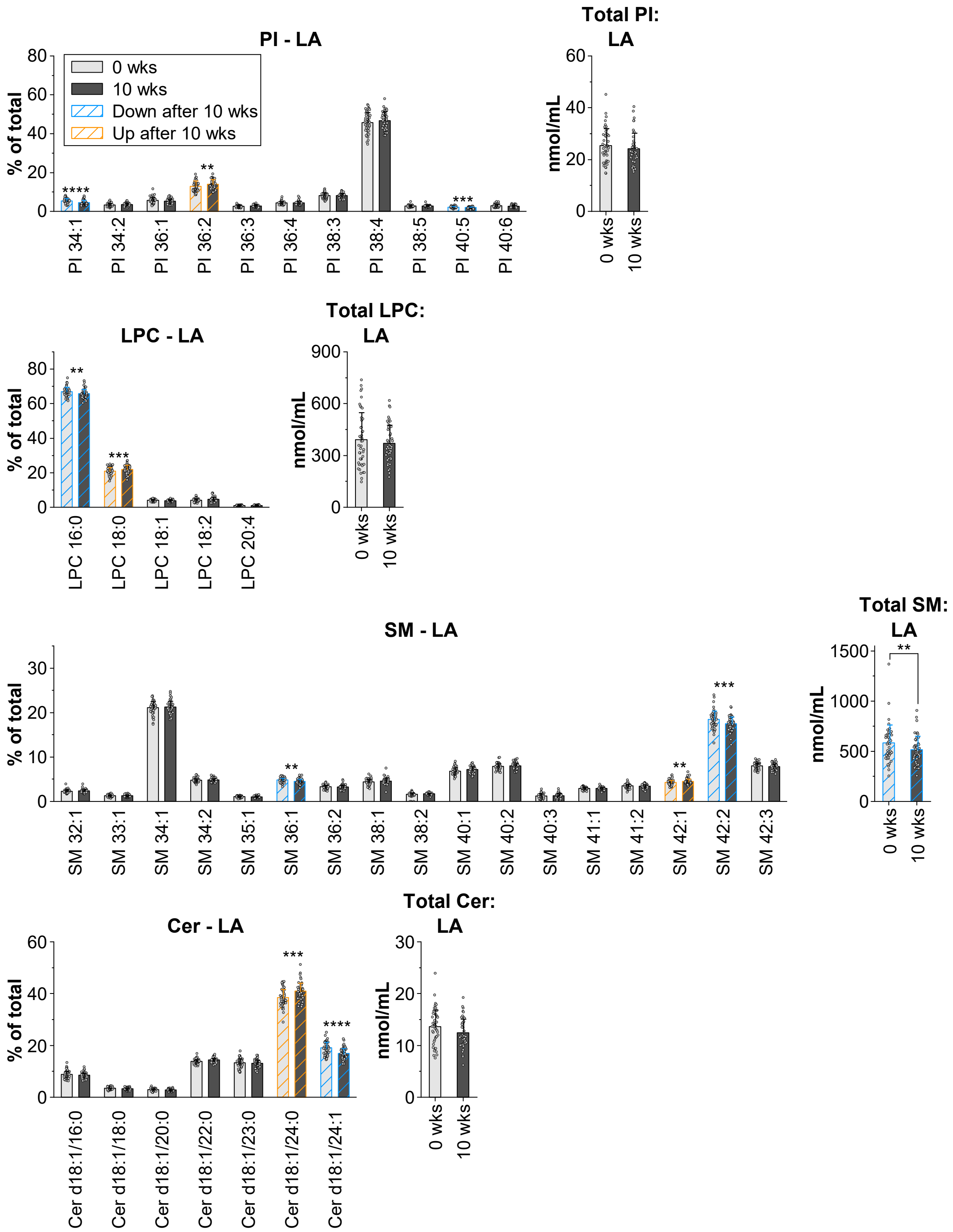

Figure S2. Impact of sunflower oil intervention on the plasma lipidome. Lipid species profiles and total lipid levels for phosphatidylinositol (PI), lysophosphatidylcholine (LPC), sphingomyelin (SM) and ceramide (Cer). Shown are means ± SD of lipid species with an average contribution > 1% from $n = 45$. ** $p < 0.01$, *** $p < 0.001$, **** $p < 0.0001$.

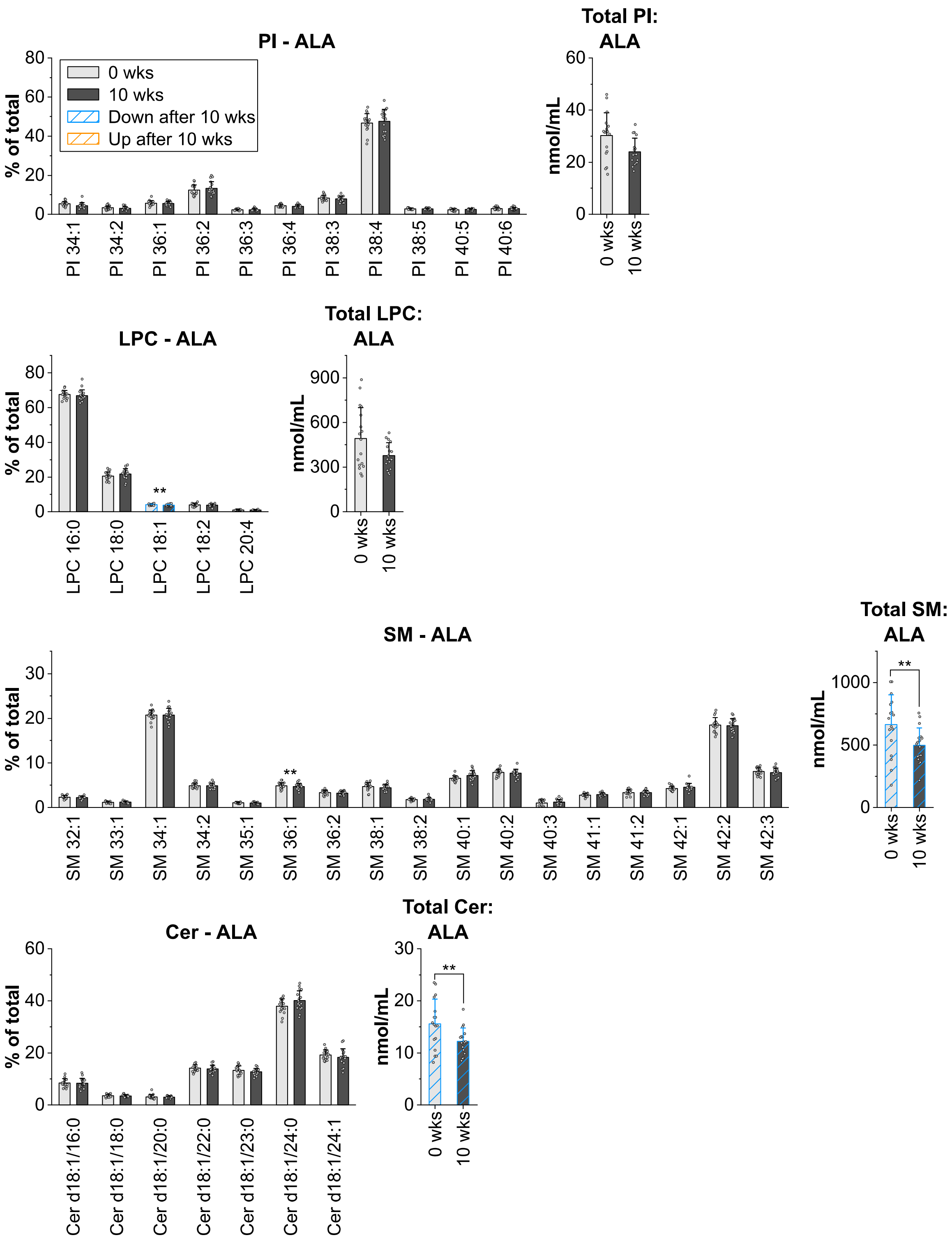

Figure S3. Impact of linseed oil intervention on the plasma lipidome. Lipid species profiles and total lipid levels for phosphatidylinositol (PI), lysophosphatidylcholine (LPC), sphingomyelin (SM) and ceramide (Cer). Shown are means ± SD of lipid species with an average contribution > 1% from $n = 17$. ** $p < 0.01$, *** $p < 0.001$, **** $p < 0.0001$.

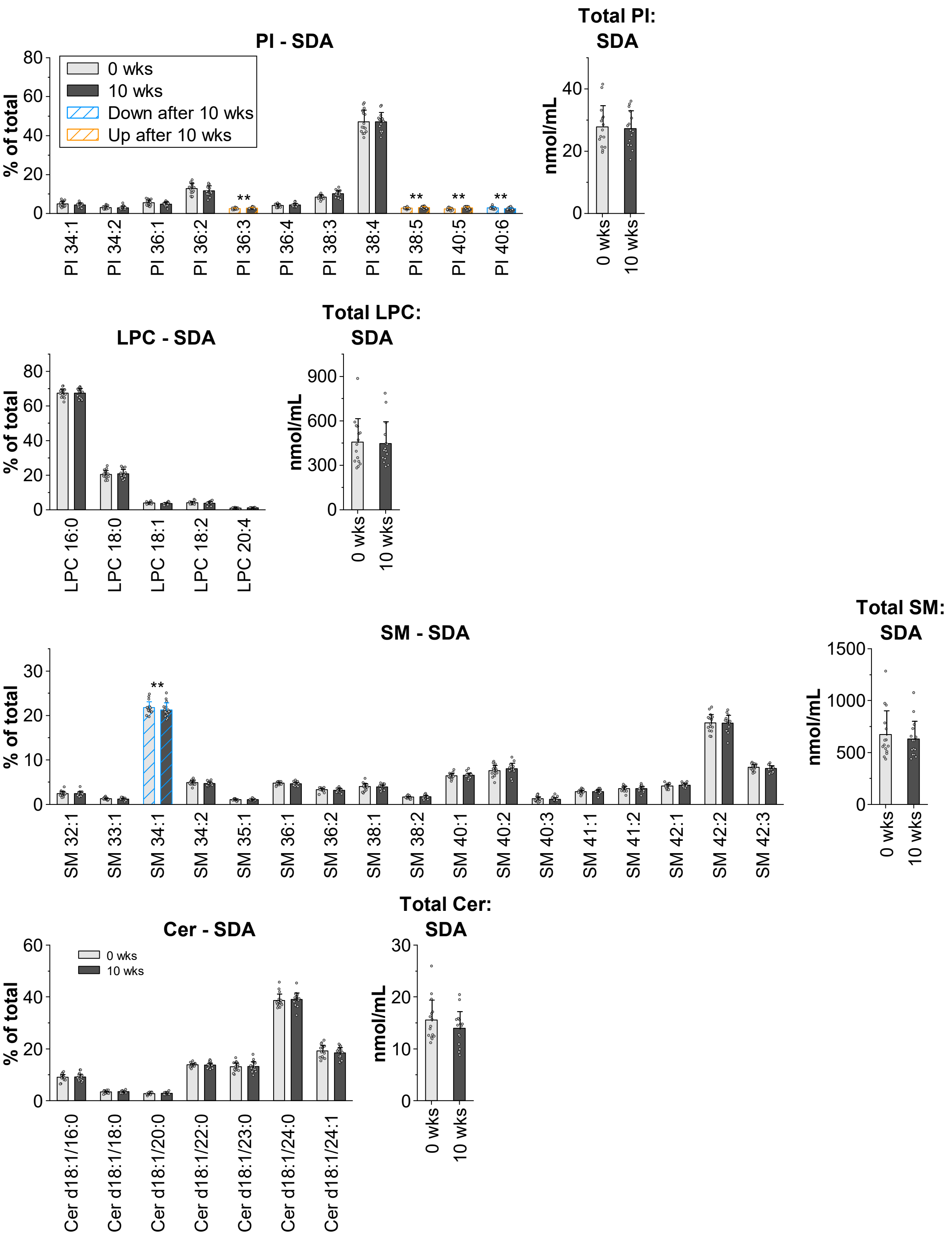

Figure S4. Impact of echium oil intervention on the plasma lipidome. Lipid species profiles and total lipid levels for phosphatidylinositol (PI), lysophosphatidylcholine (LPC), sphingomyelin (SM) and ceramide (Cer). Shown are means ± SD of lipid species with an average contribution > 1% from $n = 15$. ** $p < 0.01$, *** $p < 0.001$, **** $p < 0.0001$.

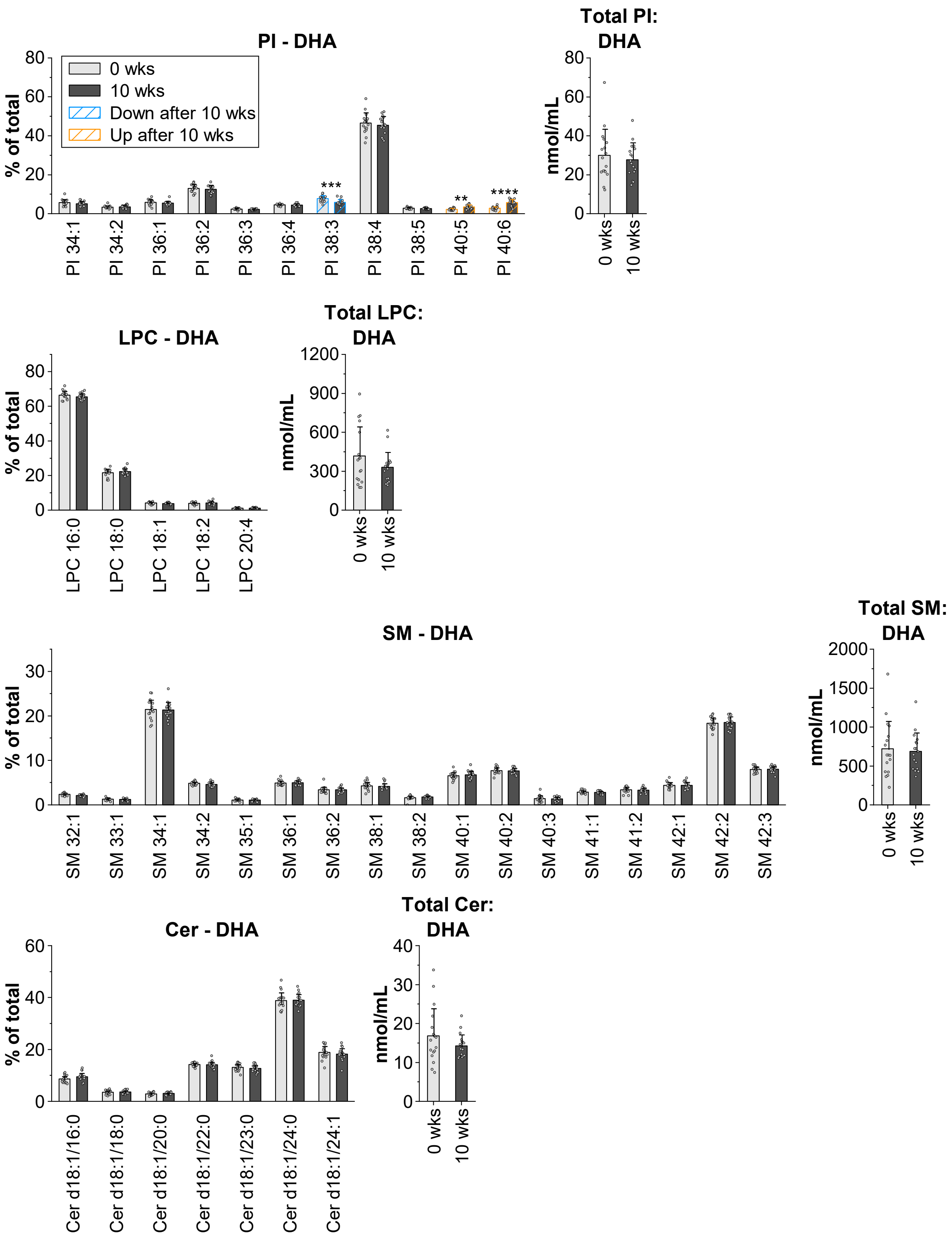

Figure S5. Impact of microalgae oil intervention on the plasma lipidome. Lipid species profiles and total lipid levels for phosphatidylinositol (PI), lysophosphatidylcholine (LPC), sphingomyelin (SM) and ceramide (Cer). Shown are means ± SD of lipid species with an average contribution > 1% from $n = 17$. ** $p < 0.01$, *** $p < 0.001$, **** $p < 0.0001$.
